# ASSOCIATION BETWEEN STATE AND TRAIT ANXIETY AND CARDIOVASCULAR DISEASE

**DOI:** 10.1093/geroni/igac059.3049

**Published:** 2022-12-20

**Authors:** Ruifeng Cui, Montgomery Owsiany, Alaa Shalaby

**Affiliations:** VA Pittsburgh Healthcare System, Morgantown, West Virginia, United States; West Virginia University, Morgantown, West Virginia, United States; VA Pittsburgh Healthcare System, Pittsburgh, Pennsylvania, United States

## Abstract

Cardiovascular disease (CVD) consistently ranks among the top ten leading causes of death, with prevalence rates being the highest among middle aged and older adults. Research has found that CVD has a negative bidirectional association with anxiety. However, the literature is limited regarding what specific aspects of anxiety, i.e., state and/or trait, are associated with CVD. The present study investigated the association between state and trait anxiety and CVD in a national sample of 640 middle aged and older adults. Participants (M age = 56.2, SD = 11.8) were from the Midlife in the United States (MIDUS) Biomarker sub-project. State anxiety was assessed with the Mood and Anxiety Symptom Questionnaire-anxiety subscale and trait anxiety was assessed with the Spielberger Trait Anxiety Inventory. In a binary logistic regression model including both state and trait anxiety, only trait anxiety significantly predicted presence of CVD (p = .05). In particular, trait anxiety was significantly associated with circulatory disease (p = .05), heart murmurs (p = .03), and blood clots (p = .03). Among middle-aged and older adults, trait anxiety is associated with having several different CVDs. These findings support assessing and addressing trait anxiety among middle aged and older adults with CVD.

